# CREATING A SUCCESSFUL DATA PLAN: NIA BEHAVIORAL AND SOCIAL AGING RESEARCH DMS REVIEW EXPERIENCE

**DOI:** 10.1093/geroni/igae098.4328

**Published:** 2024-12-31

**Authors:** James McNally, Paavani Jain

**Affiliations:** University of Michigan, Ann Arbor, Michigan, United States; National Institute on Aging, Baltimore, Maryland, United States

## Abstract

The revised NIH Data Management and Sharing (DMS) Policy took effect in January 2023, requiring all researchers applying for NIH funding to develop a detailed DMS plan as part of the application. In turn, NIH became responsible for reviewing and approving the submitted plan before issuing an award and monitoring compliance. Having recently completed our first year of DMS reviews, this presentation will report lessons learned by the NIA Division of Behavioral and Social Research (DBSR). One of the critical changes was the requirement for a six-element document addressing Data Type; Related Tools, Software, and Code; Standards; Data Preservation, Access, and Associated Timelines; Access, Distribution, or Reuse Considerations; and Oversight of Data Management and Sharing study. Despite efforts to educate and prepare all parties for this transition, researchers had questions about the best way to prepare a DMS plan and from NIA staff on reviewing the submitted requests. Within DBSR, the Office of Data Resources and Analytics (ODRA) established a review team to examine DMS plans, develop necessary resources, and coach grantees. The presentation will present lessons learned for best practices in DMS development. We will discuss handling primary versus secondary data, metadata, and source code, selecting an appropriate repository for socio-behavioral and interdisciplinary aging data, data restrictions, and navigating various grant mechanisms and their data prospects. A non-compliant DMS plan can delay or reverse funding decisions; NIA seeks to ensure all researchers understand this plan’s importance and how to successfully create one that supports ongoing scientific discovery.

